# Pancreatic acinar cell carcinoma with predominant extension into the main pancreatic duct: A case report

**DOI:** 10.1002/deo2.96

**Published:** 2022-02-24

**Authors:** Takuya Ishikawa, Eizaburo Ohno, Yasuyuki Mizutani, Tadashi Iida, Hiroki Kawashima

**Affiliations:** ^1^ Department of Gastroenterology and Hepatology Nagoya University Graduate School of Medicine Aichi Japan; ^2^ Department of Endoscopy Nagoya University Hospital Aichi Japan

**Keywords:** contrast‐enhanced endoscopic ultrasonography, endoscopic retrograde pancreatography, main pancreatic duct, pancreatic acinar cell carcinoma, pancreaticoduodenectomy

## Abstract

A 34‐year‐old male was referred to our hospital for a possible pancreatic mass detected by computed tomography (CT) that was performed to find the cause of acute pancreatitis. Multiple imaging tests, including contrast‐enhanced CT scan, magnetic resonance imaging, contrast‐enhanced endoscopic ultrasonography, and endoscopic retrograde pancreatography, revealed a solid mass occupying the head of the main pancreatic duct (MDP), and pancreaticoduodenectomy was performed. In the resected specimen, the tumor showed expansive growth from the pancreatic parenchyma to the MDP and formed a tumor plug. Histopathological findings together with immunostaining findings led to the diagnosis of pancreatic acinar cell carcinoma (PACC). The patient was alive and recurrence‐free for 11 years after surgery. Extension into the MDP is more common in PACC than in conventional pancreatic ductal adenocarcinoma. PACC patients with MDP extension may have less aggressive clinicopathologic characteristics, and a relatively good prognosis can be expected.

## INTRODUCTION

Pancreatic acinar cell carcinoma (PACC) is a rare exocrine malignancy with an incidence of less than 1% of all pancreatic exocrine neoplasms.^1^ Typically, PACC is a solid tumor that shows expansive rather than infiltrative growth, and involvement of the biliary or pancreatic duct is rare. Several reports have described an intraductal variant of PACC showing growth of the tumor predominantly within pancreatic ducts.^2^, ^3^ However, the clinicopathological features of PACC with intraductal extension have not yet been investigated. Here, we report a case of PACC with predominant extension into the main pancreatic duct (MPD) that showed characteristic findings on various imaging modalities and achieved long‐term recurrence‐free survival for more than 10 years.

## CASE REPORT

**FIGURE 1:**
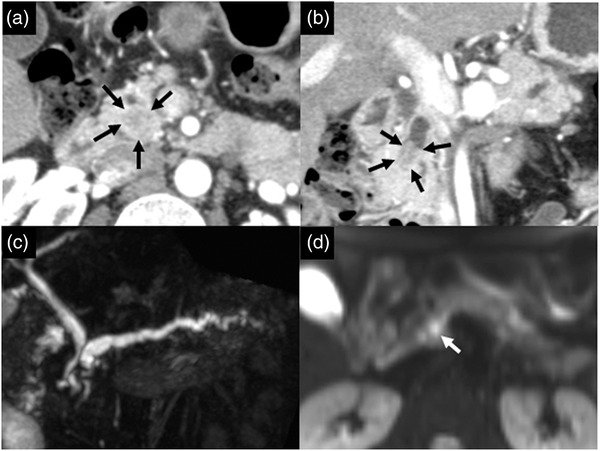
(a, b) Multiphase dynamic computed tomography (CT) scan (a) axial view, (b) coronal view showing a nodule mildly stained at the pancreatic parenchymal phase in the main pancreatic duct of the head of the pancreas (arrows). (c) Magnetic resonance cholangiopancreatography showing the poorly delineated main pancreatic duct in the head of the pancreas with upstream dilation. (d) Diffusion‐weighted image showing an increased signal in the pancreatic head (arrow)

A 34‐year‐old male was referred to our hospital because of a suspected pancreatic mass observed by computed tomography (CT), which was performed when he was hospitalized for mild acute pancreatitis. On physical examination, no mass was palpable in his abdomen. He was a social drinker and had no family history of cancer. Laboratory tests at the time of referral showed that serum amylase and lipase levels were within normal limits, and there was no elevation in the tumor markers of carcinoembryonic antigen and carbohydrate antigen 19‐9. A multiphase dynamic CT scan showed a nodule mildly stained from the pancreatic parenchymal phase to the portal phase in the MPD of the head of the pancreas (Figure 1A,B). Magnetic resonance cholangiopancreatography (MRCP) showed that the head of the MPD was poorly delineated and that the upstream MPD was dilated (Figure 1C). The T2‐weighted magnetic resonance imaging showed a low‐signal area with indistinct borders occupying the lumen of the poorly delineated area on MRCP. Diffusion‐weighted images showed an increased signal in this area (Figure 1D). At this point, we suspected that a neoplastic lesion developed in the MPD, but the possibility of pancreatic ductal invasion due to a lesion developed from pancreatic parenchyma was also considered. As a differential, intraductal lesions, such as MPD‐type intraductal papillary mucinous neoplasms or intraductal tubulopapillary neoplasms, and extraductal lesions, such as pancreatic cancer or neuroendocrine neoplasms, were listed.

**FIGURE 2:**
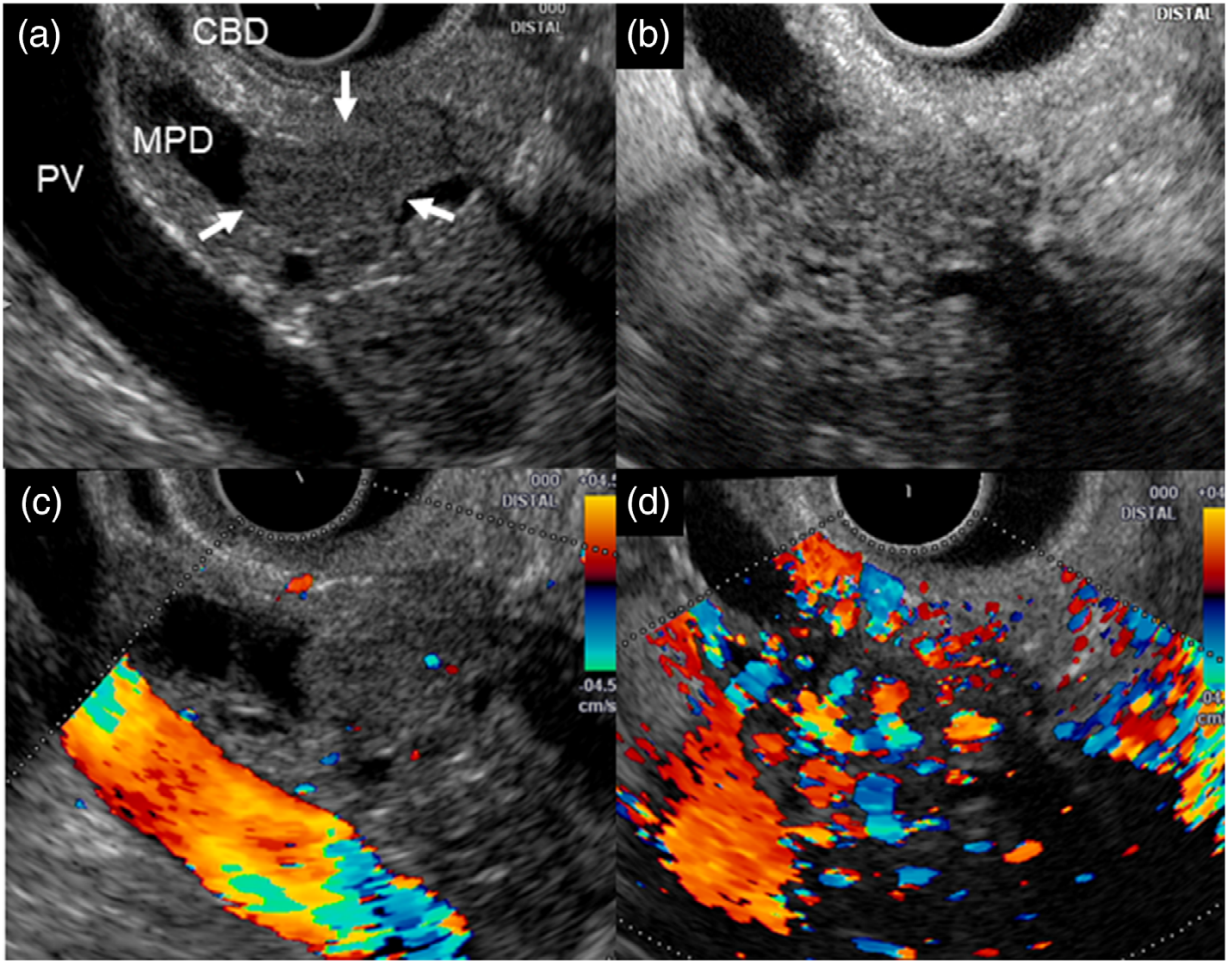
(a) Endoscopic ultrasonography (EUS) showing an isoechoic mass of 12 mm in size (arrows) in the main pancreatic duct of the head of the pancreas, and the caudal duct was markedly dilated. The common bile duct was located close to the mass, but there was no dilatation. (b) Contrast‐enhanced harmonic EUS using Sonazoid showing rapid enhancement at an early stage (20 s). (c, d) Colour doppler mode imaging (c) before Sonazoid injection, (d) 60 s after Sonazoid injection showing abundant blood flow signals in the mass after Sonazoid injection. PV: portal vein, CBD: common bile duct, MPD: main pancreatic duct

Endoscopic ultrasonography (EUS) showed an isoechoic mass of 12 mm in size in the MPD of the head of the pancreas (Figure 2A). Contrast‐enhanced harmonic EUS using Sonazoid (GE Healthcare Japan, Tokyo, Japan), which was continuously observed for 60 s (Video 1), showed an inflow of contrast medium into the mass from the early stage, and the contrast effect persisted for 60 s (Figure 2B). Colour Doppler mode (60 s after Sonazoid injection) also showed abundant blood flow signals in the mass, suggesting a hypervascular tumor (Figure 2C,D). Since the lesion was considered to be predominantly located inside the pancreatic duct, EUS‐guided fine needle aspiration (EUS‐FNA) was not performed due to the concern of pancreatitis.

**FIGURE 3:**
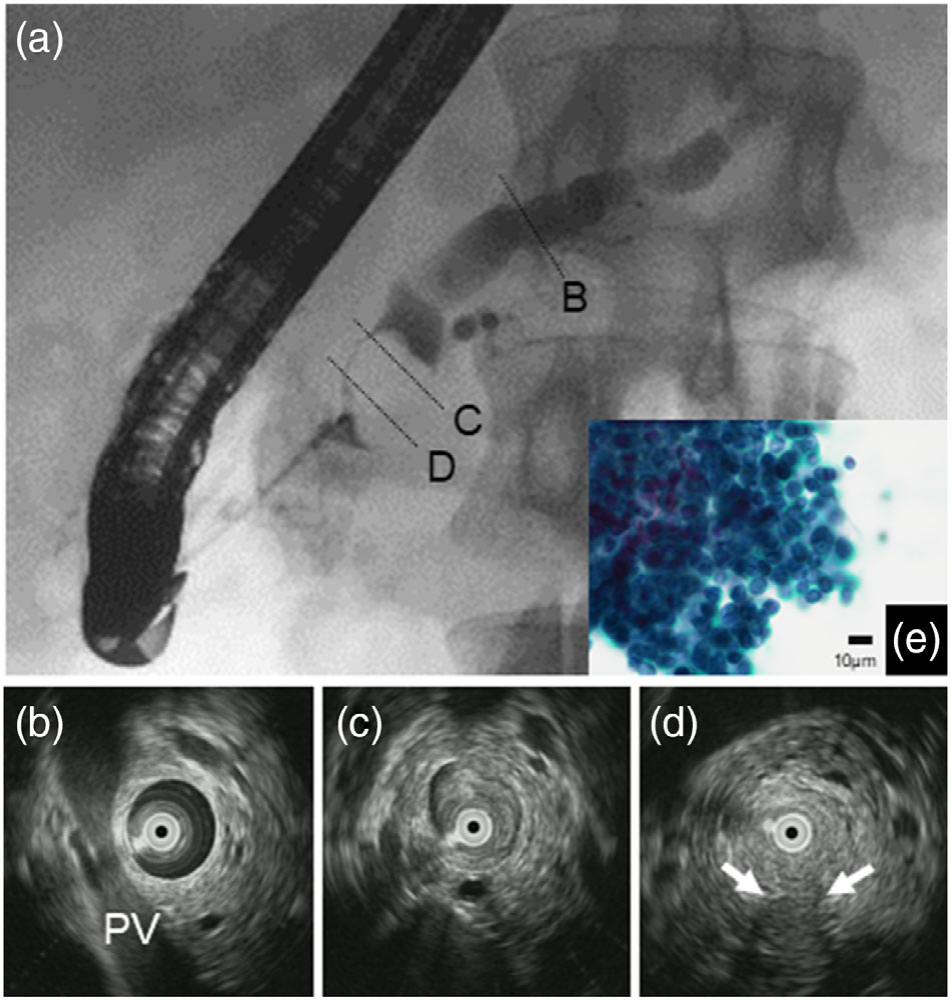
(a) Endoscopic retrograde pancreatography showing a defect with a smooth surface in the main pancreatic duct (MPD) of the head of the pancreas. (b–d) Intraductal ultrasonography showing an isoechoic mass located predominantly in the MPD and partial infiltration into the pancreatic parenchyma (arrows). (e) Pancreatic juice cytology showing a superimposed "islet‐like" conglomerate composed of small cells, which was atypical for a normal pancreatic ductal carcinoma. PV: portal vein

Endoscopically, no abnormalities were found in the duodenal major papilla, and there was no apparent mucus discharge. Endoscopic retrograde pancreatography (ERP) showed a defect with a smooth surface in the MPD of the head of the pancreas (Figure 3A). An intraductal ultrasonography (IDUS) depicted the lesion as an isoechoic mass that was friable by contact with the probe. The lesion was located predominantly in the MPD and appeared to be partially infiltrating into the pancreatic parenchyma (Figure 3B–D). Pancreatic juice cytology performed at ERP revealed the diagnosis of ‘adenocarcinoma’ (Figure 3E). However, it showed a superimposed "islet‐like" conglomerate composed of small cells, which was atypical for a normal pancreatic ductal carcinoma. Since the lesion did not spread horizontally as an IPMN and was located predominantly in the MPD, ITPN was the primary preoperative differential diagnosis. Thus, subtotal stomach‐preserving pancreaticoduodenectomy was performed.

**FIGURE 4:**
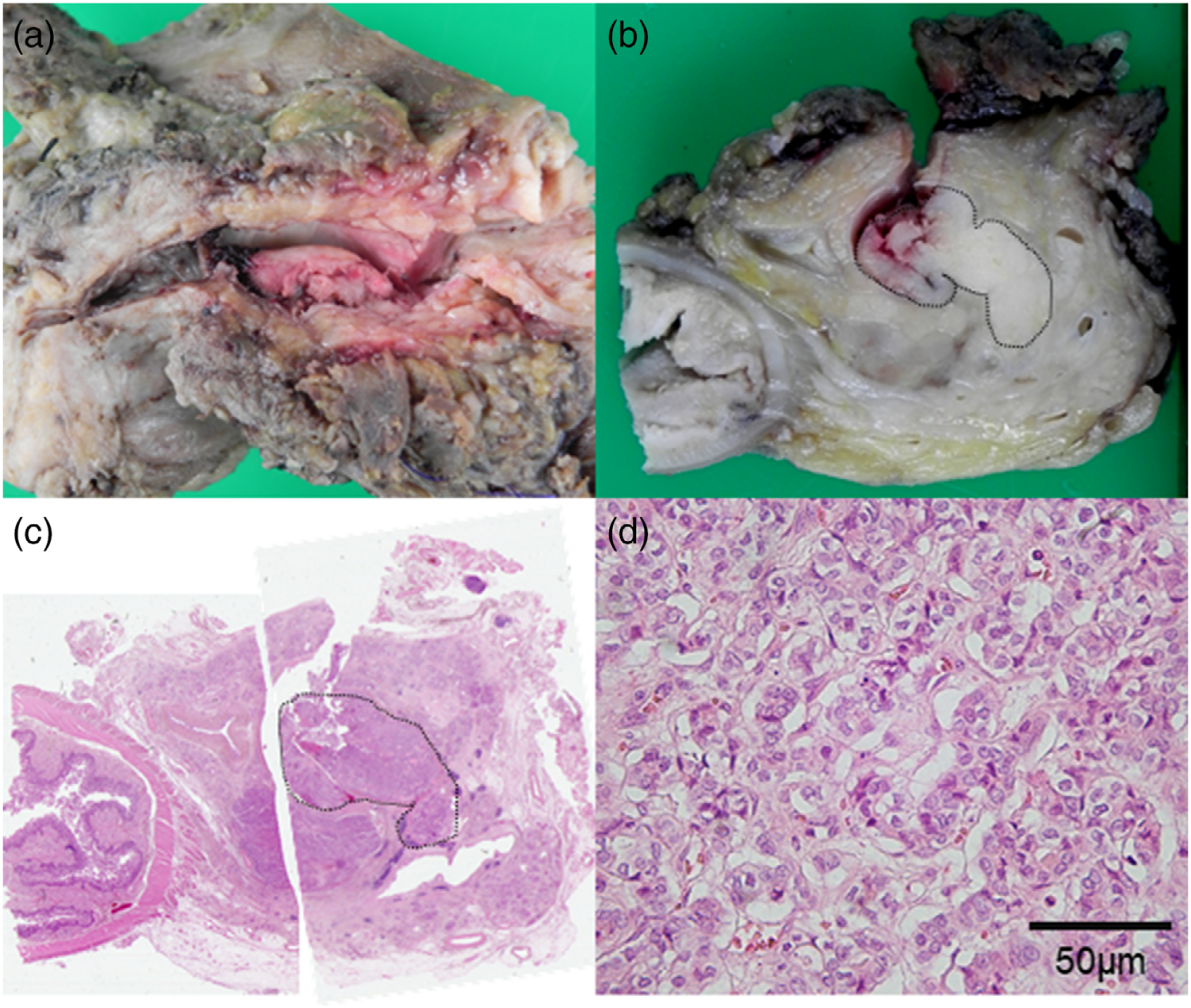
(a) Gross specimen with a split along the main pancreatic duct. (b) Cut surface of the resected specimen showing a whitish tumor present from the pancreatic parenchyma to the main pancreatic duct and forming a tumor plug in the main pancreatic duct (surrounded by a dotted line). (c) Loupe imaging of the pancreatic head showing the tumor located within the pancreas with relatively clear borders (surrounded by a dotted line). (d) Histopathological examination (H&E staining) showing dense proliferation of cells with atypical nuclei with increased chromatin and clear eosinophilic sporophytes

Macroscopically, on the resected specimen, the tumor was whitish. It was present from the pancreatic parenchyma to the MPD and formed a tumor plug in the MPD (Figure 4A,B). The tumor was located within the pancreas with relatively clear borders. Histopathological examination revealed a dense proliferation of cells with atypical nuclei with increased chromatin and clear eosinophilic sporophytes (Figure 4C,D). No lymphatic, venous, or perineural invasion was evident, and there were no lymph node metastases. The results of the immunohistochemical staining were as follows: α1‐antitrypsin (+), lipase (+), EMA (+), chromogranin A (‐), synaptophysin (‐), serotonin (‐), and Ki‐67 (3%–4%). These histopathological findings together with immunostaining led to the diagnosis of PACC. This patient was periodically followed up at our hospital and has been recurrence‐free for 11 years since surgery.

## DISCUSSION

PACC with MPD involvement was first reported in 2001,^3^ followed by several case reports of PACC with a similar progression pattern.^4^, ^5^ Moreover, the Japan Pancreas Society has stated that 29.2% of PACC cases that were entered in the Pancreatic Cancer Registry showed ‘spread within the MPD’.^6^ However, many aspects of the relationship between the pancreatic duct system and the tumor growth and extension of PACC including morphological and pathological features are still unclear. Bhosale et al.^7^ reported that 10% (3/30) of PACC cases had pancreatic ductal ingrowth, which showed a papillary pattern similar to that of IPMN.^2^ Ban et al. reported the gross and histological features of 13 cases of PACC, of which seven (54%) showed intraductal polypoid growth (IPG).^8^ In Comparison with PACC without IPG, PACC with IPG had fewer infiltrative features, including lymphatic, venous, and neural invasion, formation of tumor thrombus in the portal vein, nodal metastasis, and invasion beyond the pancreas to the surrounding organs. These findings suggest that a significant proportion of PACC shows IPG, which is potentially associated with less aggressive clinicopathologic characteristics.

Although EUS has high spatial resolution and is considered to be useful in the diagnosis of pancreatic diseases, there have been few reports on EUS findings of PACC, especially on contrast‐enhanced EUS. We previously reported a case series of five patients with PACC who received surgical treatment and compared the preoperative contrast‐enhanced EUS findings with the surgical specimens.^9^ Although they demonstrated various patterns of enhancement depending on the pathologic phenotype, such as the amount of fibrosis and invasion of the surrounding tissue, four of five (80%) cases showed vascular tumors with gradual enhancement. In the present case, the lesion was depicted as an isoechoic mass occupying the MPD on EUS, and the vascular findings on contrast‐enhanced harmonic EUS helped differentiate it from conventional pancreatic ductal adenocarcinoma.^10^ ERP also showed a unique finding of a defect with a smooth surface inside the MPD. Based on pancreatography, we suspected a tumor originating in the MPD, but IDUS indicated the tumor was partially extending into the pancreatic parenchyma.

Recently, the usefulness of EUS‐FNA for the diagnosis of PACC with MPD extension has been reported.^4^ In the present case, EUS‐FNA was not performed because of the concern of pancreatitis. However, considering the possibility that the treatment for acinar cell carcinoma may change in the future including drug therapy, it may become more important to collect tissue at the time of diagnosis.

In summary, we reported a case of PACC that initially presented with acute pancreatitis and showed predominant intraductal extension into the MPD. Extension into the MPD is more common in PACC than in conventional pancreatic ductal adenocarcinoma. Patients with MPD extension may have a lower tendency to invade than those without extension, and a relatively good prognosis can be expected.

## CONFLICT OF INTEREST

The authors declare that there are no conflicts of interest that could be perceived as prejudicing the impartiality of the research reported.

## ETHICS STATEMENT

All procedures followed have been performed in accordance with the ethical standards laid down in the 1964 Declaration of Helsinki and its later amendments. Informed consent was obtained from the patient for publishing this case report.

## FUNDING INFORMATION

None.

## Supporting information


**Video 1**. Contrast‐enhanced harmonic endoscopic ultrasonography using Sonazoid (5–15 s after injection). The inflow of contrast medium into the mass was detected from the early stage, and the contrast effect persisted for 60 s.
